# Immune Cell Landscape in Gastric Cancer

**DOI:** 10.1155/2021/1930706

**Published:** 2021-01-09

**Authors:** Yang Yang, Wei He, Zi-rui Wang, Yu-jiao Wang, Lan-lan Li, Jian-zhong Lu, Yan Tao, Jing Zhang, Sheng-jun Fu, Zhi-ping Wang, Shan-hui Liu

**Affiliations:** ^1^The Second Hospital of Lanzhou University, Lanzhou University, Lanzhou, 730000 Gansu, China; ^2^The Second Clinical Medical College of Lanzhou University, Lanzhou University, Lanzhou, 730000 Gansu, China; ^3^Department of Medical Oncology, Cancer Center, West China Hospital, Sichuan University, Chengdu, 610041 Sichuan, China; ^4^Institute of Urology, Lanzhou University Second Hospital, Lanzhou, 730000 Gansu, China; ^5^Key Laboratory of Urological Disease of Gansu Province, Lanzhou, 730000 Gansu, China; ^6^Institute of Gansu Nephro-Urological Clinical Central, Lanzhou, 730000 Gansu, China

## Abstract

**Background:**

The tumor-infiltrating immune cells are closely associated with the prognosis of gastric cancer (GC). This article is aimed at determining the composition change of immune cells and immune regulatory factors in GC and normal tissues, depicting their prognosis value in GC, and revealing the relationship between them and GC clinical parameters.

**Methods:**

We used CIBERSORT to calculate the proportion of 22 immune cells in the GC or normal tissues; a *t*-test was applied to assess the expression difference of immune cells and immune regulatory factors in normal and GC tissues. The relationship of the immune cells, immune regulatory factors, and GC patients' clinical characteristics was assessed by univariate analysis.

**Results:**

In this study, we found that the proportion of macrophages increased, while plasma cells and monocytes decreased in GC tissues. In these immune fractions, Tregs and naïve B cells were found to be correlated with GC patients' prognosis. Interestingly, the expression of immune regulatory factors was ambiguous with their classical function in GC tissues. For example, TIM-3, FOXP3, and CMTM6 were overexpressed, while CD27 and PD-1 were underexpressed in GC tissues. We also found that IDO1, PD-1, TIGIT, and TIM-3 were highly expressed in high-grade GC tissues, the HERC2 expression level was related to patients' gender, and the TIGIT expression level was sensitive to targeted therapy. Furthermore, our results suggested that the infiltration of Tregs and naive B cells was strongly correlated with the T stage, radiation therapy, targeted molecular therapy, and the expression levels of TIM-3 and FOXP3 in GC.

**Conclusion:**

The expression pattern of tumor-infiltrating immune cells and immune regulatory factors was systematically depicted in the GC tumor microenvironment, indicating that individualized treatment based on the tumor-infiltrating immune cells and immune regulatory factors may be beneficial to GC patients.

## 1. Introduction

Gastric cancer (GC) is one of the most common gastrointestinal malignancies with high morbidity and mortality [1]. Although current traditional methods (such as surgery, chemotherapy, and radiotherapy) have a curative effect for GC patients, more than 50% of GC patients relapse from these treatments and GC further develop into metastatic gastric carcinoma [2, 3]. Therefore, clarifying the tumorigenesis mechanism and finding a new treatment target and effective prognosis indexes are urgent. Immune infiltration plays an important role in GC tumorigenesis [4], and tumor immunotherapy has been paid more attention and has been used in clinical trials for GC [5, 6].

Cancer immunotherapy is aimed at improving the immune system's ability to eliminate cancer cells. Several types of immunotherapies have been developed to improve the immune system [7, 8]. Early immunotherapy is mainly focused on activating nonspecific immune cells such as NK cells and dendritic cells by cytokine stimulation [9]. However, these cells are poorly specific to tumor cells. So its clinical application was restricted. In recent years, with the further understanding of the character of T cells and B cells in the tumor environment, immunotherapy has been developed into activating the adaptive immune cells and it has shown a great advantage [10]. In particular, numerous research signs of progress have been achieved on chimeric antigen receptor T cells [11]. Besides, strategies for enhancing immune checkpoint blockade therapy, such as PD-1/PD-L1/TIM-3 inhibitors and monoclonal antibodies, also showed great potential in tumor immunotherapy [12, 13].

Due to the highly complex composition of the tumor microenvironment, the immune cells and immune regulatory factors have not been systematically studied in GC tissues. The relationship between immune cells and clinical-pathological parameters and immune regulatory factors also need to be explored. The Cancer Genome Atlas (TCGA) program, a landmark cancer genomics program, was designed to molecularly characterize over 20,000 primary cancer and matched normal samples [14]. Benefiting from the bioinformatics tool, CIBERSORT, we could systematically analyze the tumor-infiltrating immune cells in the tumor environment [15].

In this study, we calculated the proportion of 22 types of tumor-infiltrating immune cells and evaluated the expression of immune regulatory factors in human GC tissues and normal tissues. The correlation between immune cells, clinical-pathological parameters, and immune regulatory factors was also assessed. By systematically analyzing the prognosis value of the tumor-infiltrating immune cells and immune regulatory factors in GC, we hope this work would be helpful for us to understand the GC tumor environment and would be useful for GC clinical immunotherapy.

## 2. Results

### 2.1. Overview of Clinical Data of 256 Patients

In this article, we have collected 256 patients' clinical and pathological information (Table 1).

### 2.2. Adaptive Immune Cells in Gastric Cancer

**Figure 1:**
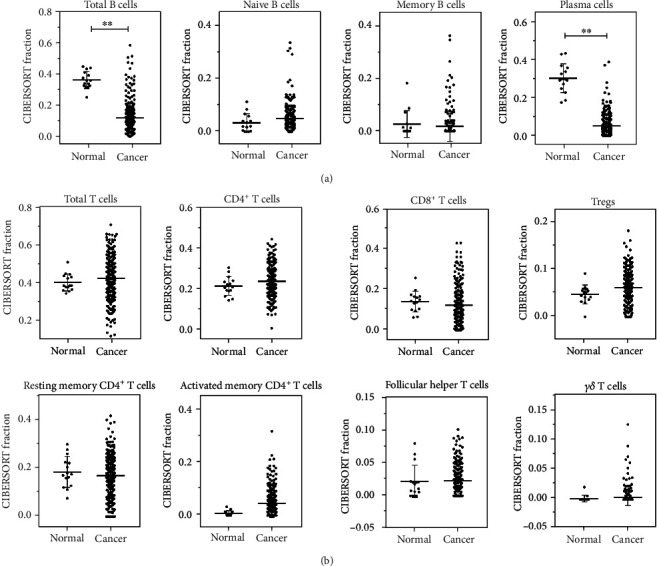
Adaptive immune cells in human GC tissue and healthy gastric tissue. (a) Total B cells, naïve B cells, memory B cells, and plasma cells and (b) total T cells, CD4^+^ T cells, CD8^+^ T cells, Tregs, resting memory CD4^+^ T cells, activated memory CD4^+^ T cells, follicular helper T cells, and *γδ* T cells were calculated for each patient group and compared using *t*-test analysis. ^∗^*P* < 0.05; ^∗∗^*P* < 0.01.

In normal and GC tissues, the fraction of naïve B cells slightly increased in GC although there was no significant difference. In GC, total B cells (95% CI -0.293–-0.190; *P* < 0.0001) and plasma cells (95% CI -0.282–-0.220; *P* < 0.0001) were significantly decreased (Figure 1(a)).

Like naïve B cells, CD4^+^ T cells were slightly higher in GC than in normal tissues although there was no significant difference. CD8^+^ T cells and resting memory CD4^+^ T cells decreased in GC tissues. In contrast, the fractions of Tregs and activated memory CD4^+^ T cells (95% CI 0.011–0.065; *P* = 0.01) were significantly higher in GC than in normal tissues, especially the activated memory CD4^+^ T cells (Figure 1(b)).

### 2.3. Innate Immune Cells in Gastric Cancer

**Figure 2:**
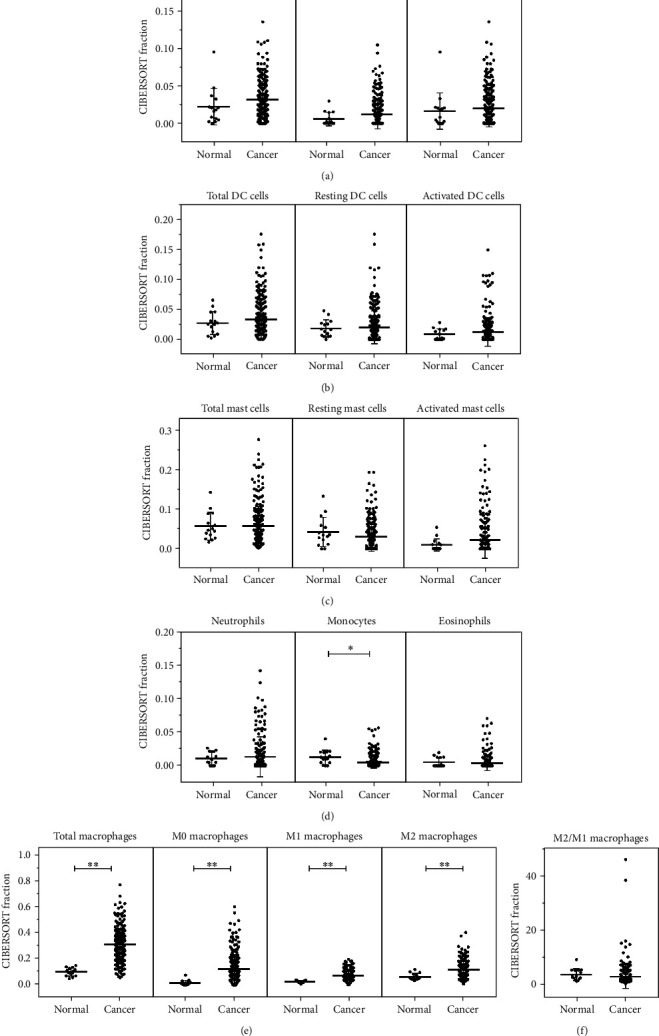
Innate immune cells in human GC tissue and healthy gastric tissue. Total nonspecific immune response cells in human GC and normal gastric tissues. (a) Total NK cells, resting NK cells, and activated NK cells; (b) total dendritic cells, resting dendritic cells, and activated dendritic cells; (c) total mast cells, resting mast cells, and activated mast cells; (d) neutrophils, monocytes, and eosinophils; (e) total macrophages, M0 macrophages, M1 macrophages, and M2 macrophages; and (f) M2/M1 macrophages were calculated for each patient group and compared using *t*-test analysis. ^∗^*P* < 0.05; ^∗∗^*P* < 0.01.

In GC tissues, the innate immune cell fractions, such as total NK cells, resting NK cells, and activated NK cells, increased (Figure 2(a)). Besides, the fractions of total DC cells, resting DC cells, and activated DC cells were also overexpressed in GC tissues (Figure 2(b)). The expression fraction of activated mast cells was higher in GC tissues although the slight fraction change showed no significant difference (Figure 2(c)). There was no significant change of the eosinophil and neutrophil fraction. Monocytes (95% CI -0.012–-0.001; *P* = 0.02) were significantly reduced in GC (Figure 2(d)). Total macrophage fraction (95% CI 0.145–0.272;*P* < 0.0001) was significantly highly expressed in GC tissues. The fractions of M0 (95% CI 0.050–0.163; *P* = 0.0003), M1 (95% CI 0.027–0.070; *P* < 0.0001), and M2 macrophages (95% CI 0.021–0.087; *P* = 0.0014) were higher in GC tissues. M2/M1 macrophages in GC tissues slightly decreased, but there was no significant change (Figures 2(e) and 2(f)).

### 2.4. Correlation with 22 Immune Cells in Gastric Cancer

**Figure 3:**
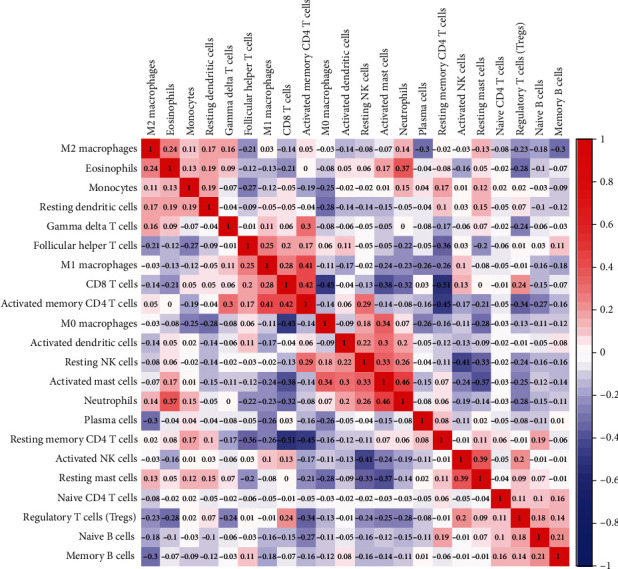
The relationship between 22 immune cells in GC. The red squares represent a positive correlation between the two immune cells, and the blue squares represent a negative correlation between the two immune cells. The numerical size represents the correlation. The larger the number, the stronger the correlation.

Based on the fraction proportion of the tumor-infiltrating immune cells, we calculated the correlation between the fractions of 22 immune cells with the corrplot R package (Figure 3). Results showed that a positive correlation existed between activated mast cells and neutrophils. The activated memory CD4^+^ T cells showed a strong positive correlation with CD8^+^ T cells and M1 macrophages. The fraction of resting memory CD4^+^ T cells negatively correlated with activated memory CD4^+^ T cells and CD8^+^ T cells. A negative correlation also existed in CD8^+^ T cells and M0 macrophages.

### 2.5. Prognostic Evaluation of Tumor-Infiltrating Immune Cells

**Figure 4:**
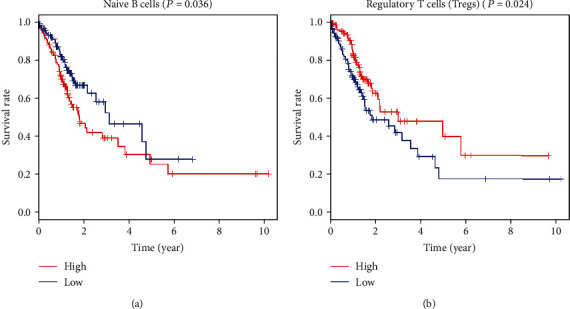
Kaplan-Meier curves and overall survival of high-infiltrating (a) naïve B cells and (b) Tregs in GC patients.

Based on the expression of tumor-infiltrating immune cells, we divided these GC patients into the high-infiltrating immune group and low-infiltrating immune group. Kaplan-Meier survival analysis was performed to analyze the tumor-infiltrating immune cell prognosis value. Results showed that patients with low-infiltrating naïve B cells (Figure 4(a)) and high-infiltrating Tregs (Figure 4(b)) had a better prognosis.

### 2.6. Immune Regulatory Factor Expression in GC Tissues and Normal Tissues

**Figure 5:**
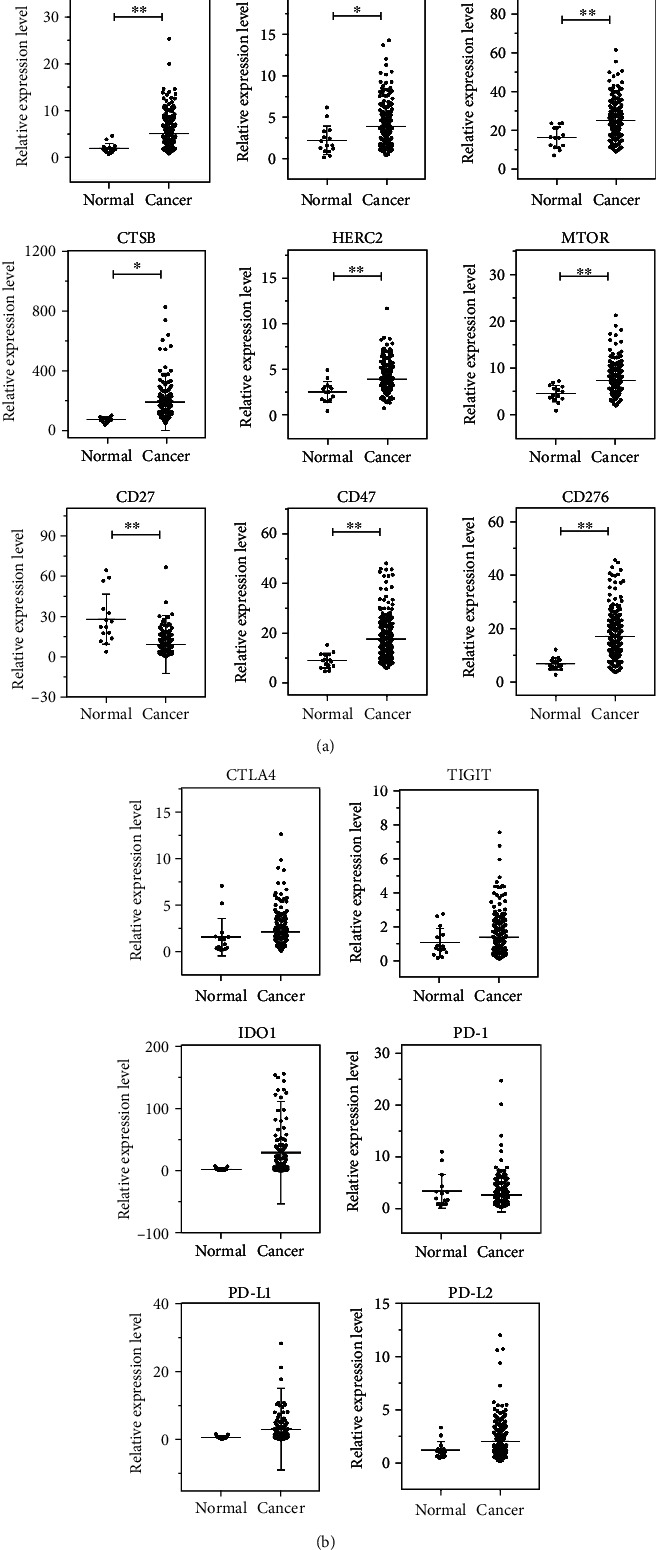
Immune regulatory factor expression in human GC and normal gastric tissues: (a) TIM-3, FOXP3, CMTM6, CTSB, HERC2, MTOR, CD27, CD47, and CD276; (b) CTLA4, TIGIT, IDO1, PD-1, PD-L1, and PD-L2. ^∗^*P* < 0.05; ^∗∗^*P* < 0.01.

Considering that immune regulatory factors play a vital role to modulate the tumor-infiltrating immune cell function, we investigated the expression of many important immune regulatory factors in GC tissues and normal tissues. As shown in Figure 5(a), the expression levels of TIM-3 (95% CI 1.341–4.781; *P* = 0.0005), FOXP3 (95% CI 0.384–2.948; *P* = 0.0111), CMTM6 (95% CI 2.80–14.94; *P* = 0.0044), CTSB (95% CI 18.51–213.5; *P* = 0.0199), HERC2 (95% CI 0.563–2.183; *P* = 0.0010), MTOR (95% CI 1.131–4.35; *P* = 0.0009), CD47 (95% CI 4.255–12.97; *P* = 0.0001), and CD276 (95% CI 5.941–14.36; *P* < 0.0001) were significantly higher in GC tissues than in normal gastric tissues. In contrast, CD27 (95% CI -30.11–-7.72; *P* = 0.0010) showed high expression in normal tissues. In addition, as shown in Figure 5(b), we found that the expression levels of CTLA4, TIGIT, IDO1, PD-L1, and PD-L2 were higher in GC, and PD-1 expression was slightly lower in GC tissues.

### 2.7. Correlation between Tumor-Infiltrating Immune Cells, Immune Regulatory Factors, and Clinical Pathological Parameters

**Figure 6:**
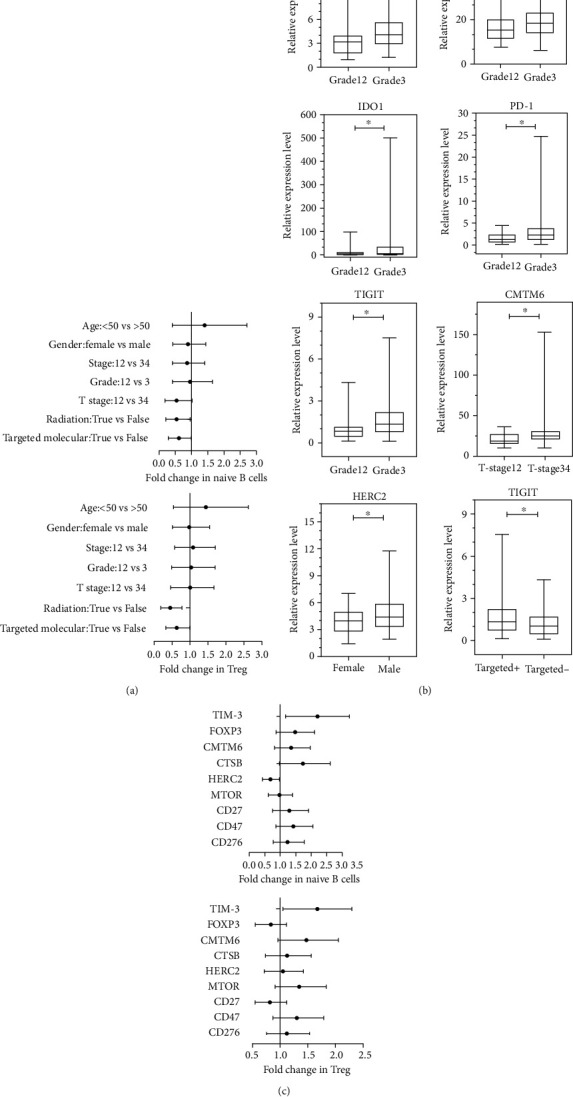
Relationship between clinical-pathological parameters and immune regulatory factors (a) and immune regulatory factors (b) in GC patients. Correlation between immune cells and immune regulatory factors (c) in GC. ^∗^*P* < 0.05; ^∗∗^*P* < 0.01.

The relationship between age, gender, clinical stage, grade, T stage, radiotherapy, and targeted molecular therapy and fractions of naïve B cells or Tregs was assessed by univariate analysis. Results suggested that naïve B cells (HR = 0.443, 95% CI 0.205–0.957, *P* = 0.038; HR = 0.542, 95% CI 0.296–0.991, *P* = 0.047) and Tregs (HR = 0.384, 95% CI 0.191–0.772, *P* = 0.007; HR = 0.575, 95% CI 0.333–0.994, *P* = 0.047) were less infiltrating in the GC patients who received radiation therapy and targeted molecular therapy (Figure 6(a)).

The relationship between clinical-pathological parameters and immune regulatory factors was assessed by *t*-test analysis. Result showed that the gene expression levels of TIM-3 (95% CI 0.298–2.277; *P* = 0.0113), CD47 (95% CI 0.510–7.738; *P* = 0.0258), IDO1 (95% CI 0.450–68.660; *P* = 0.0468), PD-1 (95% CI 0.180–2.507; *P* = 0.0241), and TIGIT (95% CI 0.144–1.171; *P* = 0.0126) increased in the high-grade group. CMTM6 (95% CI 0.379–14.810; *P* = 0.0393) also showed high expression in GC patients bearing a high stage. HERC2 (95% CI 0.061–1.444; *P* = 0.0332) expression was significantly higher in male GC patients, whereas TIGIT (95% CI -0.980–-0.028; *P* = 0.0383) decreased in GC patients who received targeted therapy (Figure 6(b)).

Univariate analysis was applied to analyze the relationship between the expression of TIM-3, FOXP3, CMTM6, CTSB, HERC2, MTOR, CD27, CD47, and CD276 and naïve B cells or Tregs. The results showed that infiltration of naïve B cells (HR = 2.060, 95% CI 1.276–3.325, *P* = 0.003) and Tregs (HR = 1.600, 95% CI 1.096–2.335, *P* = 0.015) increased in GC tumor tissues with high expression of TIM-3. In the GC tissues with high HERC2 expression, infiltration of naïve B cells (HR = 0.646, 95% CI 0.426–0.980, *P* = 0.040) was decreased (Figure 6(c)).

## 3. Discussion

Gastric carcinoma, a highly common malignant tumor, is mainly treated by surgery, chemotherapy, and radiotherapy. However, these treatment options always do not work for some GC patients [16]. Nowadays, targeted therapy and immunotherapy showed more attractive attention for their specific targeting ability and low toxicity, especially to advanced gastric cancer [16–18]. The GC tumor environment is highly complex and heterogeneous. Further understanding of each composition proportion and interrelationship will be helpful for GC therapy. Benefiting from TCGA database and the bioinformatics tool, CIBERSORT, we could systematically investigate the tumor-infiltrating immune cells and the expression of many key immune regulatory factors in GC tissues. This makes it possible to explore their relationship to clinical-pathological parameters and their potential prognosis value.

Herein, we investigated the fraction of tumor-infiltrating immune cells by dividing them into cellular immunity and humoral immunity components. We found that the number of CD4^+^ T cell fraction increased in GC, and CD8^+^ T cell fraction decreased. The tumor antigen-specific CD8^+^ T cells are negatively regulated by PD-1 and TIM-3 in GC [19]. Therefore, we believed that targeted therapy by increasing the level of CD8^+^ T cells would be an effective method to inhibit GC. Tregs could promote tumorigenesis and tumor immune escape by secreting immunosuppressive cytokines such as TGF-*β* which can promote the expression of antiapoptotic molecules and help tumor cells to defend against apoptosis [20, 21]. Interestingly, in our work, high Treg infiltration portended a more favorable prognosis in GC. This result was consistent with other group studies where the high expression of FOXP3, a specific transcriptional regulator in Tregs, also showed a favorable prognosis [22–24]. In addition, patients who received radiation therapy and targeted molecular therapy always often were accompanied by low Treg infiltration. Since low Treg infiltration showed a poor prognosis, improving the radiation therapy and targeted molecular therapy may be urgently needed in GC.

Tumor-associated macrophages (TAM) are the largest fraction of tumor-infiltrating immune cells in the tumor environment [25]. They could be characterized by M0 macrophages, M1 macrophages, and M2 macrophages [26]. It was always believed that M1 macrophages possessed the ability to eliminate tumor cells. In contrast to M1 macrophages, M2 macrophages could express a variety of immunosuppressive factors and chemokines, which inhibit antitumor immunity by reducing antigen presentation and inhibiting T cell function [27]. Compared to the normal tissues, the fraction proportion of M1 and M2 macrophages increased significantly in GC. M2/M1 proportion decreased slightly with no significant change [28]. Therefore, based on the different immune effects of M1 and M2 in the tumor environment, we assumed that the strategy to induce the M1 macrophage expression and inhibit the M2 macrophage expression may be also useful to improve the therapeutic effect for GC patients.

In the tumor microenvironment of gastric cancer, it was found that when mast cells were activated, they could produce and secrete neutrophil chemotactic factors, which in turn promotes an increase in the recruitment of neutrophils [29]. Other studies have shown that mast cells secrete lymphangiogenic factors to promote the formation of lymphatic vessels, which in turn promotes the production of neutrophils [30]. We found that activated mast cells were positively correlated with neutrophils. These studies are consistent with our findings.

Immune regulatory factors play a key role in tumorigenesis and in immunotherapy directly or indirectly. For example, FOXP3 is always highly expressed in the tumor environment and is capable of mediating immune escape [31, 32]. High expression of FOXP3 was correlated with a poor prognosis [33]. Our study also showed the high expression of FOXP3 in GCs, and the expression of FOXP3 is associated with the fraction of naïve B cells and Tregs in GCs. CD27/CD70 signaling promotes effector and memory T cell differentiation and enhances B cell and NK cell activation and function [34]. CD27/CD70 is expressed in CD20^+^ B cells and CD8^+^ T cells in the tumor microenvironment of gastric cancer. These cells are involved in antitumor immunity and are associated with survival in gastric cancer patients [35, 36].

In this work, we have identified and explored the expression of several key immune regulatory factors and their relationship to tumor-infiltrating immune cells, GC patients' tumor grade, tumor stage, and some other clinical parameters. Our work indicated that CD27 was underexpressed in GC. Furthermore, the expression of CD8^+^ T cells also decreased in GC tissues. As the interaction of CD27 on a T cell and CD70 on a B cell enhances T cell activation in terms of proliferation, we speculated that inducing CD27 expression may be a promising way to activate CD8^+^ T cells in GCs. TIM-3, CTLA4, and TIGIT primarily trigger peripheral tolerance and promote tumor growth by inhibiting the activity of effector T cells [37, 38]. Highly expressed TIM-3 is closely related to the poor prognosis of gastric cancer [39]. CTLA4 is an important immune checkpoint for tumor immunotherapy, such as the CTLA4 inhibitor ipilimumab, which has been tested in clinical trials in multiple types of tumors [40]. The high expression of TIGIT inhibits the antitumor function of CD8 T cells in the microenvironment of gastric cancer [41]. In our study, we did not observe a significant expression change of these genes. CD47 produces cascades that inhibit the phagocytosis of macrophages [42]. Here, we can observe that it was highly expressed in GC tissues. CD47 and TIM-3 expression levels in high-grade GC tissue were significantly increased. Interestingly, regarding the relationship between TIM-3 and GC prognosis, we found that high expression of TIM-3 shows a favorable prognosis. This result is consistent with Holderried et al.'s research [43]. Besides, we found that TIGIT expression decreases in patients who received targeted therapy.

PD-1 and PD-L1 restrict T lymphocyte antitumor function by inhibiting T cell activation [44]. Nowadays, PD-1/PD-L1 inhibitors and antibodies have been applied to clinical trials. In this work, we did not observe a significant difference change of PD-1/PD-L1 expression in GC tissues and in normal tissues. Recently, research suggested that CMTM6 could block the degradation of PD-L1 and stabilize PD-L1 [45]. Our results showed that CMTM6 increased significantly in GC and was also upregulated in GC tissues with a high stage. Therefore, we assumed that CMTM6 may be a potential target for GC immunotherapy. HERC2 has been rarely studied, and our results showed that its expression is related to gender, which is significantly higher in men. This result was supported by the epidemiology result that the incidence of GC is twofold in males than in females [46]. Therefore, HEGC2 could be regarded as a result affected by gender. It may be a reference factor for individualized or personalized GC therapy. However, due to the limited sample size of the dataset, these studies still need more samples and research evidence to validate.

Taken together, our study is the first to identify the tumor-infiltrating immune cells and immune regulatory factors with their prognosis value and their correlation with the tumor clinical index. This study indicates that tumor-infiltrating immune cells are important determinants of prognosis in GC. Meanwhile, it reveals several potential targets and biomarkers for immunotherapy development.

## 4. Materials and Methods

### 4.1. Data Collection

All data in this study were downloaded from the public database. The normal gastric tissue (*N* = 32) and GC tissue (*N* = 381) gene expression data and clinical information data were downloaded from The Cancer Genome Atlas (TCGA, https://cancergenome.nih.gov/). The corresponding clinical data, including age, gender, grade and stage, T stage, radiation therapy, targeted molecular therapy, and survival status, were also collected.

### 4.2. Immune Cell Evaluation

CIBERSORT is an analytical tool used to provide an estimation of the immune cell infiltration in a mixed cell population from their gene expression profile [47]. In this study, we used CIBERSORT to calculate the fraction of 22 infiltrating immune cells in normal gastric and GC tissues with the RNA expression data. These immune cell components included naïve B cells, memory B cells, naïve CD4^+^ T cells, resting memory CD4^+^ T cells, activated memory CD4^+^ T cells, CD8^+^ T cells, follicular helper T cells, regulatory T cells (Tregs), gamma delta (*γδ*) T cells, resting dendritic (DC) cells, activated DC cells, M2 macrophages, M1 macrophages, M0 macrophages, resting natural killer (NK) cells, activated NK cells, resting mast cells, activated mast cells, plasma cells, monocytes, eosinophils, and neutrophils. The total B cells included naïve B cells, memory B cells, and plasma cells. The total T cells included naïve CD4^+^ T cells, resting memory CD4^+^ T cells, activated memory CD4^+^ T cells, CD8^+^ T cells, follicular helper T cells, Tregs, and *γδ* T cells.

### 4.3. Data Statistics

In this work, data analysis and statistics are performed by using R. The survival time is defined from the diagnosed date to the dead time. Univariate analysis was used to assess the relationship between immune cells and clinical-pathological parameters and immune regulatory factors. A *t*-test was applied to assess the different expression levels of tumor-infiltrating immune cells and immune regulatory factors in normal and GC tissues. It was also used to analyze the clinical-pathological parameters and immune regulatory factors. *P* value < 0.05 was considered statistically significant (^∗^*P* < 0.05, ^∗∗^*P* < 0.01).

## Figures and Tables

**Table 1:** Primary tumor characteristics.

Variable	Number of samples	%	Valid (%)
Age at diagnosis			
≤50	18	7.0	7.5
>50	223	87.1	92.5
Missing	15	5.9	
Gender			
Male	93	36.3	38.0
Female	152	59.4	62.0
Missing	11	4.3	
Stage			
I	25	9.8	11.2
II	77	30.1	34.5
III	100	39.1	44.8
IV	21	8.2	9.4
Missing	33	12.9	
Grade			
G1	6	2.3	2.6
G2	67	26.2	28.5
G3	162	63.2	68.9
Missing	21	8.2	
T stage			
T1	5	2.0	2.1
T2	55	21.5	23.3
T3	110	43.0	46.6
T4	66	25.8	28.0
Missing	20	7.8	
Radiation treatment			
True	23	9.0	20.9
False	87	34.0	79.1
Missing	20	57.0	
Targeted molecular therapy			
True	51	19.9	45.9
False	60	23.4	54.1
Missing	145	56.6	

## Data Availability

Our raw data types are RNA-seq, survival data, and clinical data. All data are downloaded from the public database (TCGA database: https://cancergenome.nih.gov/). And our data does not involve animal experiments, human clinical trials, etc., so ethical approval and other content are not required.

## Abbreviations

GC: Gastric cancer
NK cells: Natural killer cells
TCGA: The Cancer Genome Atlas
Tregs: Regulatory T cells
*γδ* T cells: Gamma delta T cells
DC cells: Dendritic cells
TAM: Tumor-associated macrophages.

## Supplementary Materials

Supplementary Materials Table S1: relationship between immune cell fraction and prognosis. Table S2: relationship between immune regulatory factor fraction and prognosis. Table S3: relationship between immune regulatory factors and clinical-pathological parameters. Figure S1: analysis of the prognosis value of tumor-infiltrating immune cells. Figure S2: analysis of the prognosis value of tumor-infiltrating immune cells. Figure S3: analysis of the prognosis value of immune regulatory factors. Figure S4: analysis of the prognosis value of immune regulatory factors. Figure S5: relationship between immune regulatory factors and clinical-pathological parameters. Figure S6: relationship between immune regulatory factors and clinical-pathological parameters. Figure S7: relationship between immune regulatory factors and clinical-pathological parameters. Figure S8: relationship between immune regulatory factors and clinical-pathological parameters.
