# Supplementary material for: Immune Cell Landscape in Gastric Cancer
Source: Biomed Res Int. 2021 Jan 9;2021:1930706. doi: 10.1155/2021/1930706 (PMC7857889; doi:10.1155/2021/1930706)
Supplement: Supplementary Materials — Table S1: relationship between immune cell fraction and prognosis. Table S2: relationship between immune regulatory factor fraction and prognosis. Table S3: relationship between immune regulatory factors and clinical-pathological parameters. Figure S1: analysis of the prognosis value of tumor-infiltrating immune cells. Figure S2: analysis of the prognosis value of tumor-infiltrating immune cells. Figure S3: analysis of the prognosis value of immune regulatory factors. Figure S4: analysis of the prognosis value of immune regulatory factors. Figure S5: relationship between immune regulatory factors and clinical-pathological parameters. Figure S6: relationship between immune regulatory factors and clinical-pathological parameters. Figure S7: relationship between immune regulatory factors and clinical-pathological parameters. Figure S8: relationship between immune regulatory factors and clinical-pathological parameters. [file 1930706.f1.docx]

**TableS1.Relationship between immune cells fraction and prognosis**

| **Immune Cell** | ***P*-Value** | **Immune Cell** | ***P*-Value** |
| --- | --- | --- | --- |
| naïve B cells | 0.036 | NK cells activated | 0.644 |
| memory B cells | 0.613 | Dendritic cells resting | 0.410 |
| Plasma cells | 0.678 | Dendritic cells activated | 0.273 |
| T cells CD4^+^ naive | 0.303 | Mast cells resting | 0.629 |
| T cells CD4^+^ memory resting | 0.065 | Mast cells activated | 0.839 |
| T cells CD4^+^ memory activated | 0.106 | Macrophages M0 | 0.842 |
| T cells CD8^+^ | 0.090 | Macrophages M1 | 0.921 |
| T cells follicular helper | 0.475 | Macrophages M2 | 0.459 |
| T cells gamma delta | 0.191 | Neutrophils | 0.383 |
| T cells regulatory | 0.024 | Monocytes | 0.289 |
| NK cells resting | 0.729 | Eosinophils | 0.536 |

**TableS2.Relationship between immune regulatory factors fraction and prognosis**

| **Immune regulatory factor** | ***P*-Value** | **Immune regulatory factor** | ***P*-Value** |
| --- | --- | --- | --- |
| TIM-3 | 0.0073 | CD276 | 0.4337 |
| FOXP3 | 0.3176 | CTLA4 | 0.6408 |
| CMTM6 | 0.4395 | TIGIT | 0.8490 |
| CTSB | 0.1125 | IDO1 | 0.7908 |
| HERC2 | 0.2535 | PD-1 | 0.6724 |
| MTOR | 0.0979 | PD-L1 | 0.4971 |
| CD27 | 0.9158 | PD-L2 | 0.6809 |
| CD47 | 0.0922 |  |  |

**TableS3.Relationship between immune regulatory factors and clinical pathological parameters**

| **Immune regulatory factor** | **Age**  **≤50 vs >50** | | **Gender**  **female vs male** | | **Stage**  **12 vs 34** | | **Grade**  **12 vs 3** | | **T-stage**  **12 vs 34** | | **Radiation**  **True vs False** | | **Targeted molecular**  **True vs False** | |
| --- | --- | --- | --- | --- | --- | --- | --- | --- | --- | --- | --- | --- | --- | --- |
|  | ***P*-Value** | **95% CI** | ***P*-Value** | **95% CI** | ***P*-Value** | **95% CI** | ***P*-Value** | **95% CI** | ***P*-Value** | **95% CI** | ***P*-Value** | **95% CI** | ***P*-Value** | **95% CI** |
| TIM-3 | 0.3701 | -2.268–0.852 | 0.4370 | -0.516–1.188 | 0.1454 | -0.243–1.623 | 0.0113 | 0.298–2.277 | 0.4155 | -0.675–1.621 | 0.6189 | -1.466–0.877 | 0.5089 | -1.249–0.623 |
| FOXP3 | 0.5014 | -2.036–1.002 | 0.2133 | -0.339–1.501 | 0.0563 | -0.024–1.776 | 0.2252 | -0.380–1.593 | 0.7632 | -1.290–0.949 | 0.7299 | -1.338–0.941 | 0.6637 | -1.111–0.711 |
| CMTM6 | 0.4288 | -5.990–13.990 | 0.9457 | -5.893–6.314 | 0.4101 | -3.506–8.522 | 0.3356 | -9.684–3.335 | 0.0393 | 0.379–14.810 | 0.2025 | -2.631–12.260 | 0.3937 | -3.398–8.562 |
| CTSB | 0.7284 | -118.600-169.000 | 0.4952 | -57.270–117.600 | 0.6311 | -65.540–107.600 | 0.4793 | -127.200–60.150 | 0.3521 | -55.710–155.000 | 0.3524 | -56.770–157.800 | 0.0619 | -4.091–165.300 |
| HERC2 | 0.6153 | -0.865–1.455 | 0.0332 | 0.061–1.444 | 0.4857 | -0.452–0.944 | 0.7534 | -0.878–0.637 | 0.7929 | -0.967–0.741 | 0.2288 | -0.337–1.391 | 0.1282 | -0.156–1.219 |
| MTOR | 0.1019 | -0.351–3.825 | 0.1117 | -0.243–2.302 | 0.8757 | -1.174–1.375 | 0.9968 | -1.378–1.384 | 0.3376 | -2.302–0.797 | 0.4670 | -0.999–2.163 | 0.6470 | -0.973–1.559 |
| CD27 | 0.9631 | -16.670–17.460 | 0.1381 | -18.030–2.533 | 0.1091 | -1.879–18.410 | 0.2722 | -4.909–17.230 | 0.5326 | -8.572–16.480 | 0.6271 | -9.631–15.900 | 0.7063 | -8.268–12.160 |
| CD47 | 0.8394 | -6.259–5.095 | 0.4584 | -2.155–4.743 | 0.4002 | -1.956–4.858 | 0.0258 | 0.510–7.738 | 0.1300 | -0.952–7.304 | 0.2640 | -1.833–6.619 | 0.4294 | -4.746–2.034 |
| CD276 | 0.5877 | -6.145–3.500 | 0.2836 | -1.336–4.514 | 0.4425 | -4.027–1.773 | 0.2491 | -4.961–1.301 | 0.0999 | -6.437–0.571 | 0.0756 | -6.783–0.338 | 0.7729 | -3.313–2.470 |
| CTLA4 | 0.8753 | -1.287–1.098 | 0.3080 | -0.350–1.096 | 0.1297 | -0.163–1.257 | 0.1281 | -0.174–1.365 | 0.7673 | -1.008–0.746 | 0.4672 | -1.219–0.563 | 0.0990 | -1.296–0.113 |
| TIGIT | 0.3590 | -1.184–0.433 | 0.6808 | -0.597–0.391 | 0.0553 | -0.011–0.949 | 0.0126 | 0.144–1.171 | 0.8608 | -0.650–0.544 | 0.5313 | -0.799–0.415 | 0.0383 | -0.979–-0.028 |
| IDO1 | 0.3140 | -25.960–80.030 | 0.9202 | -30.800–34.080 | 0.9064 | -30.170–33.980 | 0.0468 | 0.500–68.660 | 0.5058 | -25.930–52.240 | 0.0992 | -6.345–72.360 | 0.4365 | -44.330–19.280 |
| PD-1 | 0.6621 | -1.423–2.231 | 0.2670 | -1.730–0.484 | 0.1194 | -0.227–1.949 | 0.0241 | 0.180–2.507 | 0.9004 | -1.260–1.430 | 0.6425 | -1.047–1.689 | 0.1228 | -1.931–0.233 |
| PD-L1 | 0.6243 | -9.227–15.300 | 0.8413 | -6.720–8.234 | 0.8832 | -6.844–7.942 | 0.9627 | -8.203–7.824 | 0.3795 | -4.993–13.000 | 0.3772 | -5.065–13.260 | 0.1248 | -12.940–1.598 |
| PD-L2 | 0.9889 | -1.041–1.027 | 0.0932 | -0.090–1.151 | 0.1548 | -0.171–1.062 | 0.0810 | -0.074–1.255 | 0.6469 | -0.584–0.936 | 0.8433 | -0.852–0.697 | 0.1432 | -1.068–0.157 |


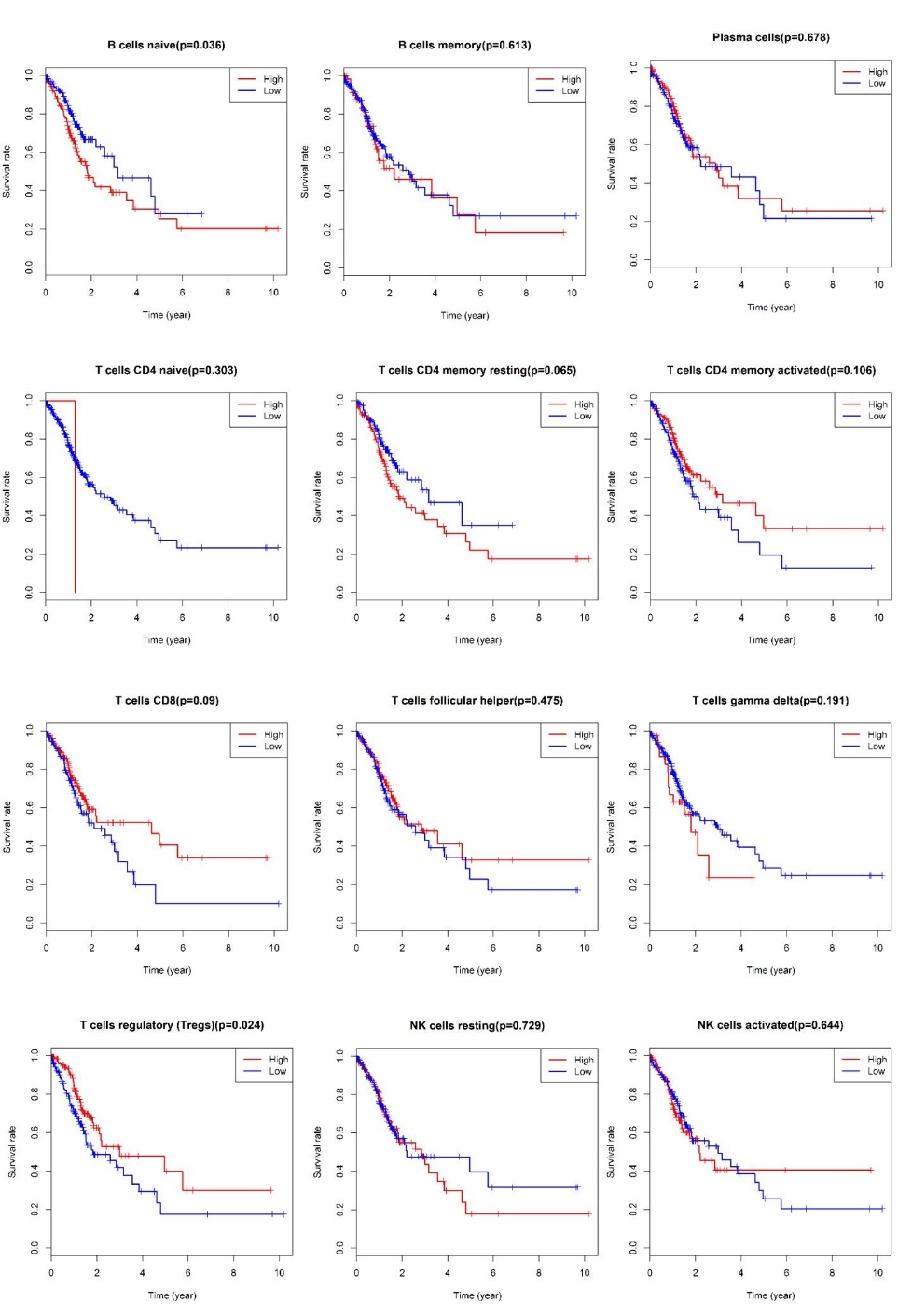


**Figure S1. Analysis of the prognosis value of tumor-infiltrating immune cells**


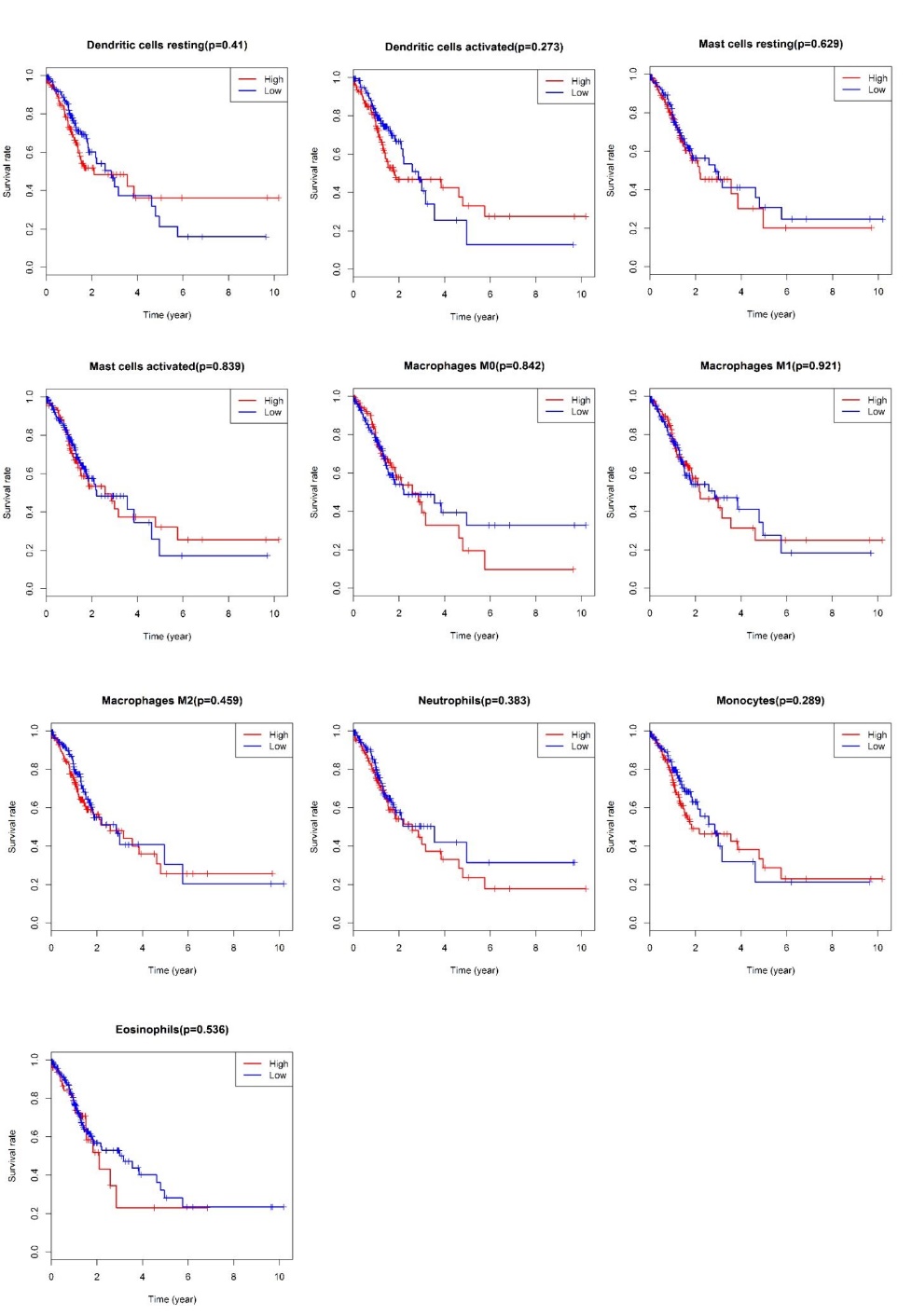


**Figure S2. Analysis of the prognosis value of tumor-infiltrating immune cells**


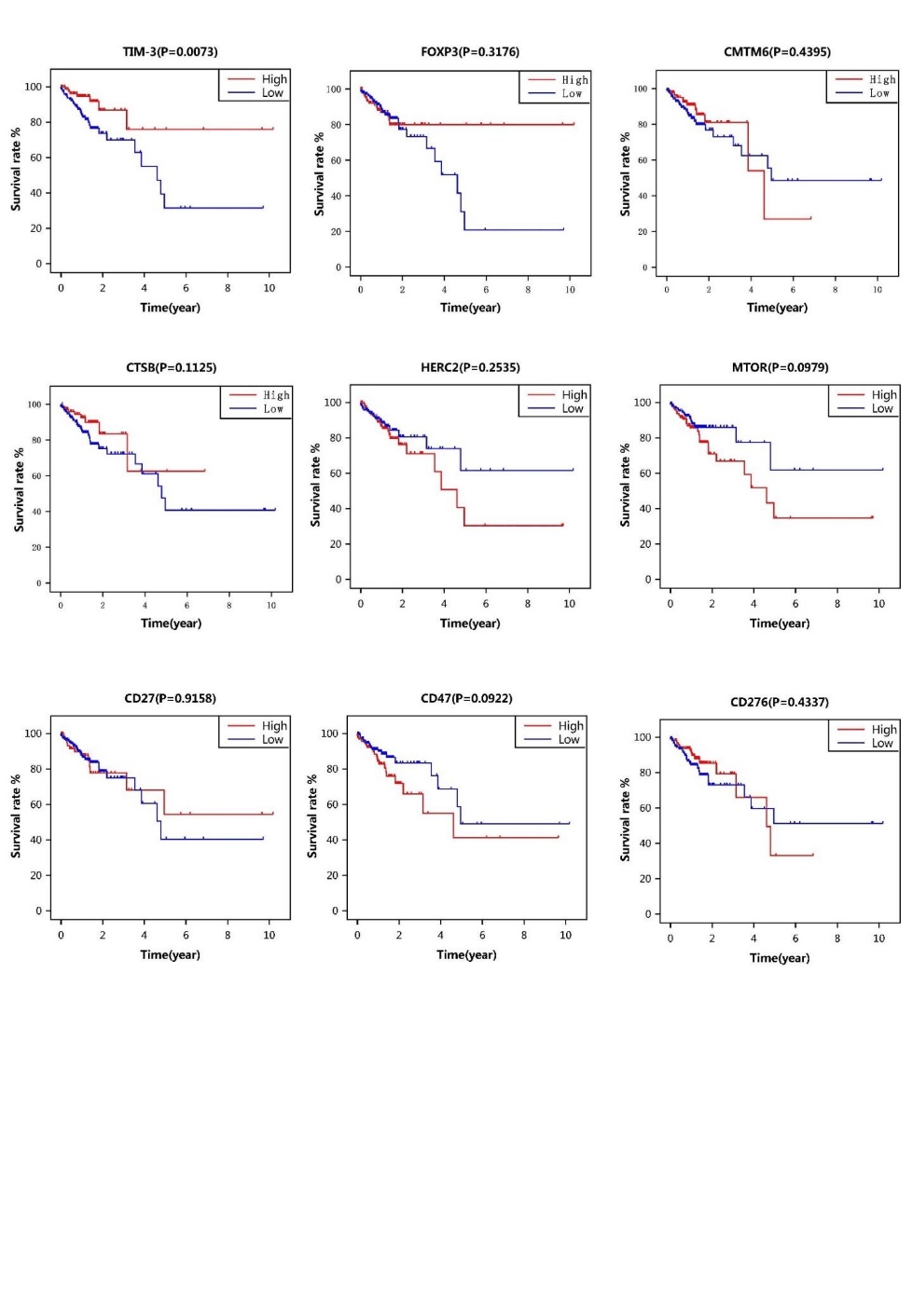


**Figure S3. Analysis of the prognosis value of immune regulatory factors**


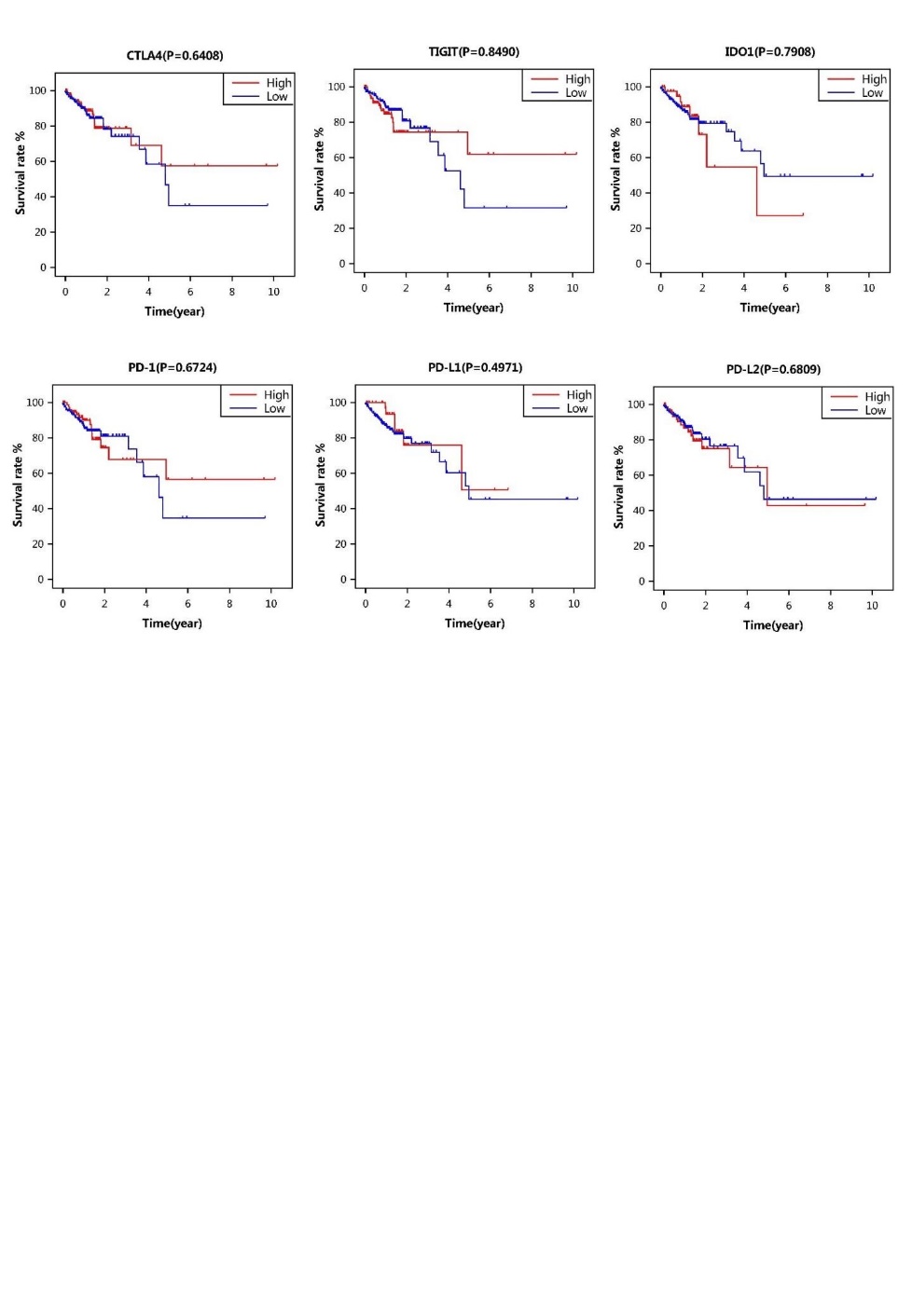


**Figure S4. Analysis of the prognosis value of immune regulatory factors**


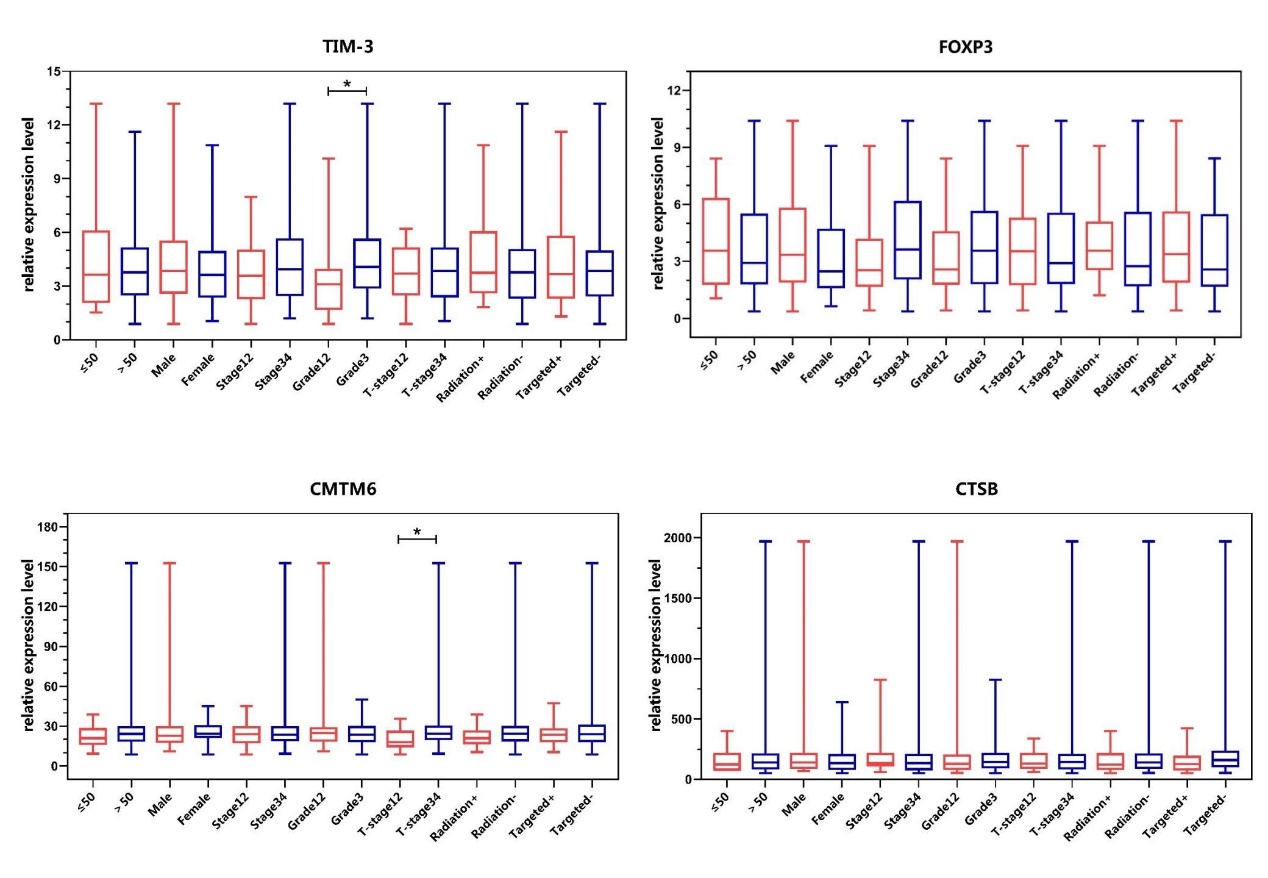


**Figure S5. Relationship between immune regulatory factors and clinical pathological parameters**


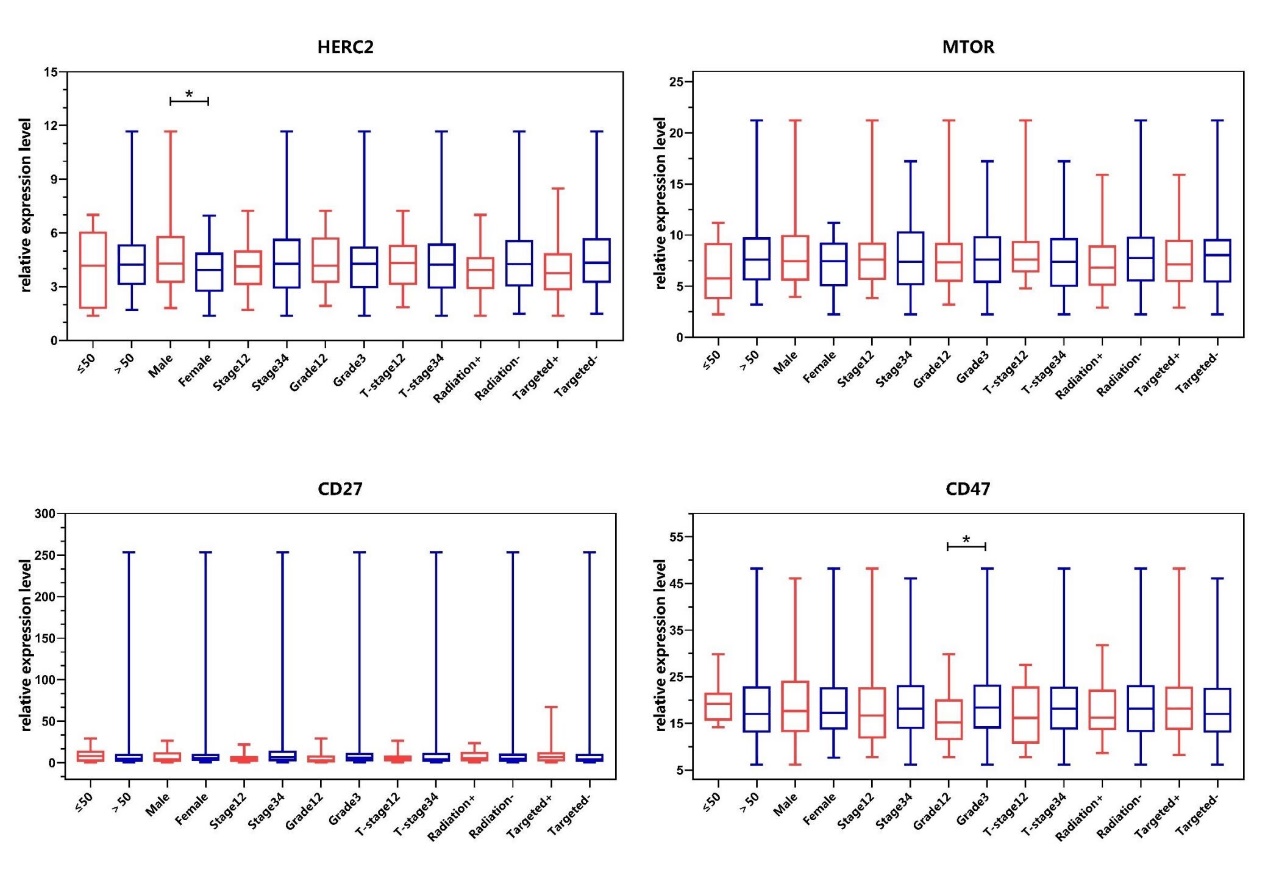


**Figure S6. Relationship between immune regulatory factors and clinical pathological parameters**


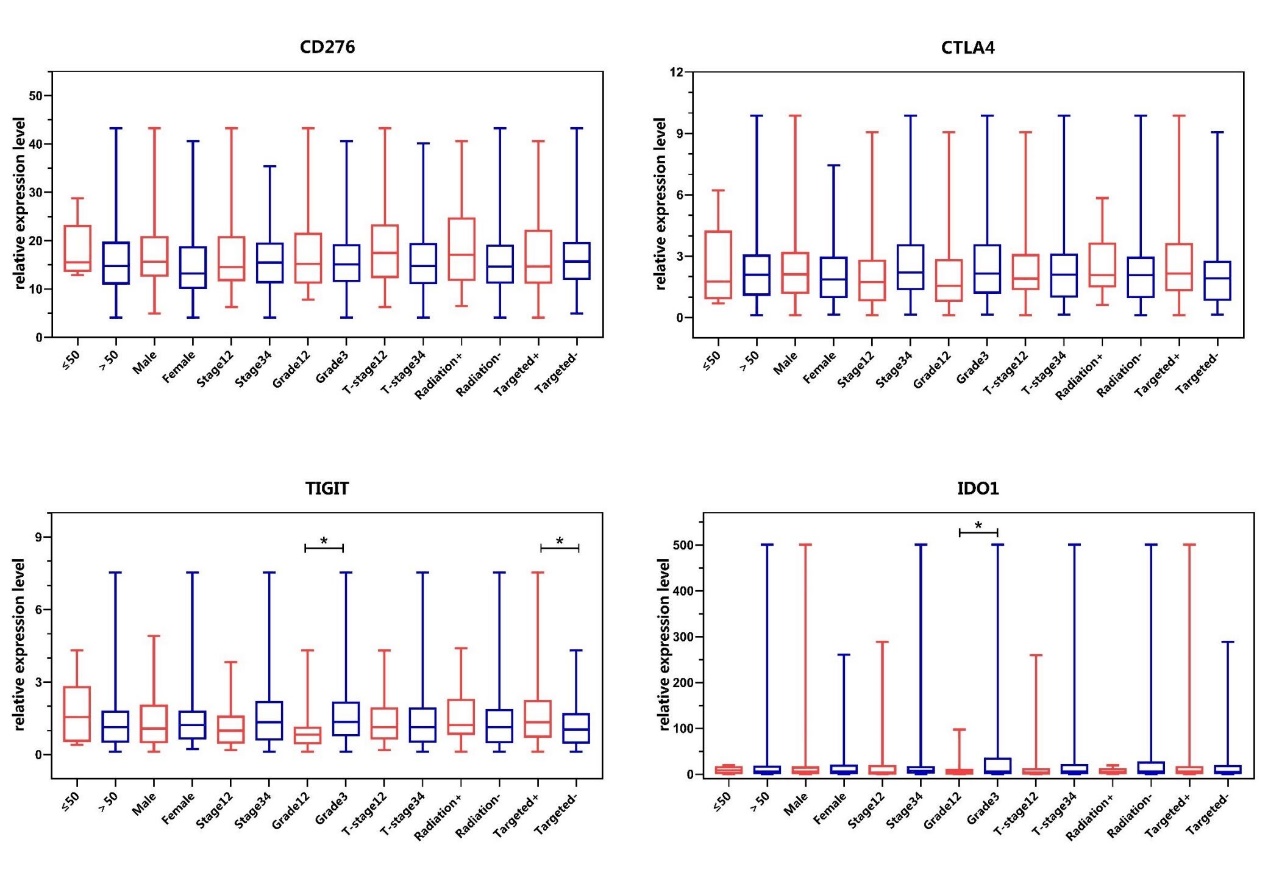


**Figure S7. Relationship between immune regulatory factors and clinical pathological parameters**


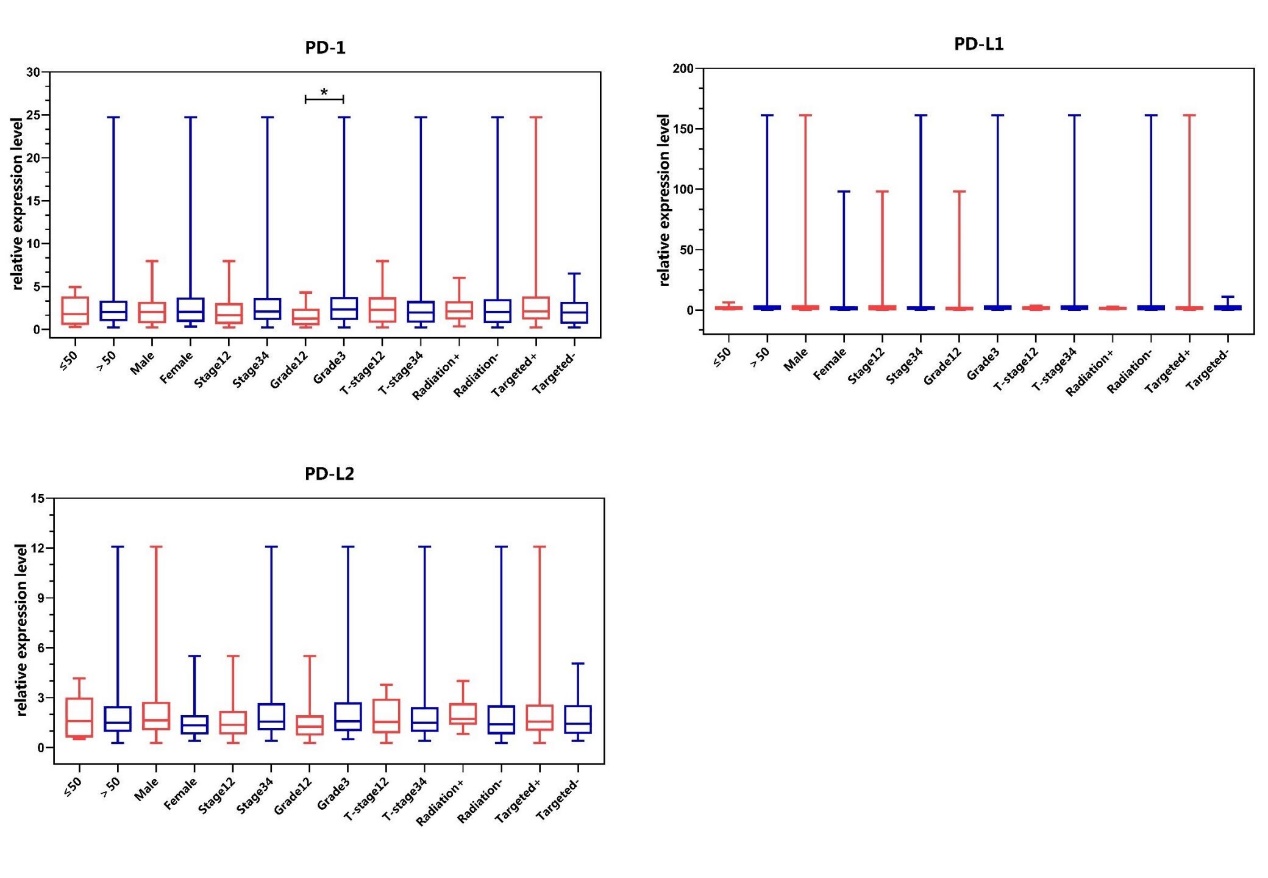


**Figure S8. Relationship between immune regurlatory factors and clinical pathological parameters**
